# Uncovering species- and drug-class-specific antimicrobial resistance mechanisms from large-scale whole-genome sequencing data using discordance analysis and machine learning

**DOI:** 10.1093/bib/bbag451

**Published:** 2026-08-27

**Authors:** Nyeong-Jin Cheon, Xuan Cuong Nguyen, Tatsuya Unno

**Affiliations:** Department of Biological Sciences and Biotechnology, Chungbuk National University, Chungdae-ro 1, Seowon-gu, Cheongju 28644, Republic of Korea; Center for Ecology and Environmental Toxicology, Chungbuk National University, Chungdae-ro 1, Seowon-gu, Cheongju 28644, Republic of Korea; Institute of Research and Development, Duy Tan University, 03 Quang Trung, Da Nang, Vietnam; Department of Biological Sciences and Biotechnology, Chungbuk National University, Chungdae-ro 1, Seowon-gu, Cheongju 28644, Republic of Korea

**Keywords:** antimicrobial resistance, whole-genome sequencing, machine learning, gene expression, genotype–phenotype discordance, random forest

## Abstract

Whole-genome sequencing has become the standard molecular platform for antimicrobial resistance surveillance, yet existing computational approaches—gene catalogs that ignore expression, *k*-mer machine learning (ML) that lacks mechanistic interpretability, and Single Nucleotide Polymorphism-based methods that capture only one resistance mechanism—suffer from systematic genotype–phenotype discordance whose molecular causes remain unquantified. We present a 16-step genomic annotation protocol that integrates promoter strength, ribosome binding site efficiency, codon adaptation, mobile element context, and chromosomal point mutations, and benchmark four ML classifiers across 20 296 bacterial genomes from five World Health Organization priority species with matched susceptibility phenotypes. Variant-specific discordance analysis revealed three categories of genotype–phenotype mismatch: antibiotics predictable by gene presence alone (tetracyclines, sulfonamides); those requiring expression context to distinguish functional from silent genes (β-lactams, aminoglycosides); and those dependent on chromosomal mutations invisible to gene catalogs (quinolones). Silent resistance genes showed 30%–85% lower predicted translation initiation rates, 4%–14% lower gene coverage, and weaker promoter activity than functional copies. Among the four classifiers evaluated, Random Forest achieved the highest performance. Expression features improved its F1 by 0.07–0.11 for β-lactams and aminoglycosides but were irrelevant for quinolones, where point mutations were essential. Mobility and regulation features were dispensable. The full model achieved mean F1 of 0.804–0.932 across species; SHapley Additive exPlanations analysis confirmed that the dominant predictive feature per drug class directly reflects its known resistance mechanism, and phylogeny-blocked cross-validation confirmed that single-gene predictions generalize across lineages. These findings establish a mechanistic framework for when and why genomic prediction fails, with direct implications for the design of clinical sequencing-based diagnostic tools.

## Graphical Abstract

**Graphical Abstract:**
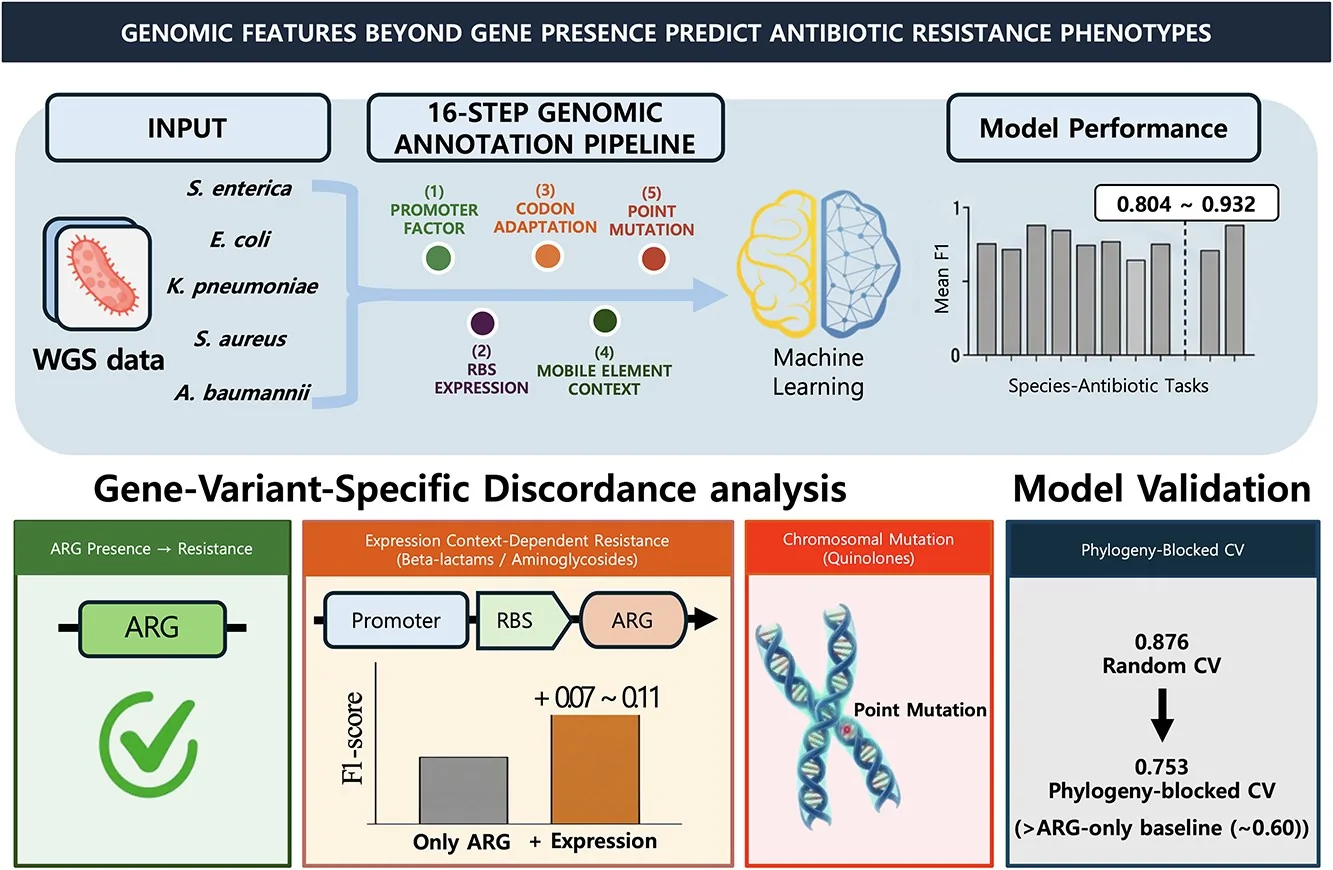

## Introduction

The relationship between antimicrobial resistance (AMR) genotype and phenotype is imperfect. While whole-genome sequencing (WGS) enables rapid identification of antimicrobial resistance genes (ARGs), the presence of an acquired ARG does not guarantee phenotypic resistance. This genotype–phenotype discordance represents a fundamental gap limiting the clinical utility of WGS-based AMR prediction.

Three biological mechanisms contribute to this discordance. First, ARGs may be “silent,” indicating that the genes are present in the genome but not producing functional protein at levels sufficient to confer resistance, due to weak promoter activity, inefficient ribosome binding, poor codon adaptation, gene truncation, or insertion sequence disruption [1]. For aminoglycosides, different modifying enzyme variants have overlapping but distinct substrate specificities, meaning gene detection alone cannot predict which antibiotics are affected [2]. Second, for several drug classes, most notably fluoroquinolones, resistance arises primarily through chromosomal point mutations (e.g. *gyrA*, *parC*) rather than acquired genes [3], making gene-catalog approaches fundamentally blind to the dominant resistance mechanism. Third, the genomic context of an ARG (e.g. location on plasmid versus chromosome, proximity to insertion sequences, and integration within mobile genetic elements) modulates its phenotypic impact through effects on gene expression [4].

Current WGS-based AMR prediction relies on two broad strategies, both with structural limitations. Gene-catalog tools including ResFinder [5], AMRFinderPlus [6], and CARD RGI [7] cannot distinguish functional from silent copies of acquired ARGs. The second strategy uses machine learning (ML) with *k*-mer or pangenome features [8, 9]; these methods achieve high accuracy but cannot explain which genomic features drive a prediction for a given drug class, although frameworks such as SHapley Additive exPlanations (SHAP) now make such feature-level attribution feasible [10]. Recent large-scale benchmarking studies have identified gene expression differences as a plausible but computationally unaddressed explanation for residual prediction failures [11], and confirmed that none of the evaluated state-of-the-art ML methods incorporate expression-level features despite their recognized potential [12].

Individual genomic context features have been explored in isolation such as insertion sequence (IS) element-driven promoters in *Acinetobacter baumannii* [13] and codon usage as a horizontal transfer indicator [14]. No study, however, has systematically quantified the contribution of gene silencing, chromosomal mutations, and expression context to genotype–phenotype discordance across diverse species and drug classes.

**Figure 1:**
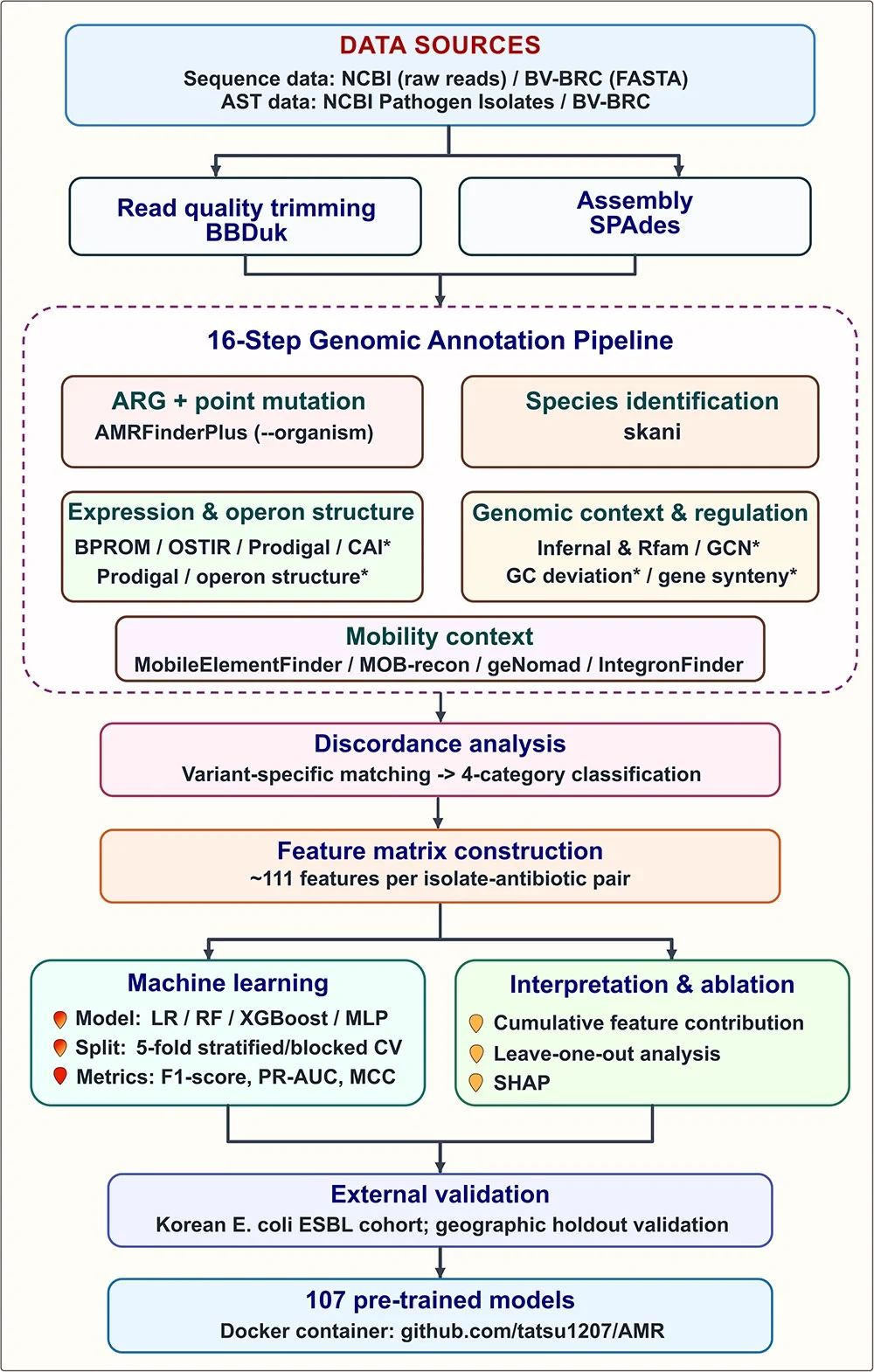
Analysis workflow; flowchart illustrating the complete pipeline from raw sequencing data through genomic annotation, feature extraction, ML model training, and evaluation; the 16-step annotation pipeline integrates tools for ARG detection, expression prediction, mobile element identification, and population structure analysis; pretrained models for all 107 species–antibiotic prediction tasks are available as a docker container.

In this study, we developed a 16-step genomic annotation pipeline (Fig. 1) that characterizes each detected ARG with features spanning transcription, translation, mobility, genomic neighborhood, and chromosomal mutations. By applying this pipeline to 20 296 genomes from five World Health Organization (WHO) priority pathogens and analyzing discordance using variant-specific matching, we identify the molecular mechanisms that explain why ARG detection is insufficient to predict phenotype and demonstrate that these mechanisms can be captured computationally from assembled genome sequences alone.

Here, we present a large-scale analysis across 107 species–antibiotic prediction tasks spanning five WHO priority pathogens, benchmarking four ML classifiers and applying variant-specific discordance analysis, cumulative feature ablation, and SHAP-based feature attribution to test four specific hypotheses. First, variant-specific substrate matching would reduce apparent genotype–phenotype discordance relative to drug-class matching. Second, expression features (promoter strength, ribosome binding site (RBS) efficiency, and codon adaptation) would resolve silent-gene discordance, with the largest gains concentrated in β-lactams and aminoglycosides. Third, chromosomal point-mutation features in *gyrA*, *parC*, and *parE* would be essential for quinolone prediction, because resistance is driven chiefly by chromosomal mutations rather than by acquired ARGs. Fourth, mobility features would add little beyond expression features because mobility affects phenotype only through expression, and regulatory-element features would also add little because their sequence-based detection is unreliable.

## Materials and methods

### Data collection and assembly

A total of 20 296 bacterial WGS datasets with associated AST phenotypes were retrieved from the NCBI Sequence Read Archive (SRA) and BioSample, and Bacterial and Viral Bioinformatics Resource Center (BV-BRC) databases, representing clinical and surveillance isolates tested against at least one antimicrobial agent. Phenotypes coded as susceptible (S), intermediate (I), or resistant (R) were binarized by grouping I with R as non-susceptible, consistent with clinical interpretation where standard-dose treatment is expected to be ineffective for both categories. Antimicrobial susceptibility phenotypes were extracted from structured BioSample metadata fields (antibiogram tables) deposited by submitting laboratories alongside genome sequences.

Raw paired-end reads were downloaded from the SRA and quality-trimmed using BBDuk (https://sourceforge.net/projects/bbmap/) to remove adapter sequences and low-quality bases. Trimmed reads were assembled de novo using SPAdes [15], producing contiguous assemblies for downstream annotation. Species identification was performed on all assemblies using skani v0.2+ against the Genome Taxonomy Database release 10 [16], based on ANI. The dataset spans five WHO priority species dominated by Enterobacterales (Salmonella, E. coli, Klebsiella) from surveillance programs (NARMS, Global Antimicrobial Resistance and Use Surveillance System (GLASS)) and including critical-priority pathogens (A. baumannii, K. pneumoniae).

### Inclusion criteria

For each species, an antibiotic was included for analysis if at least 30 isolates were present in the minority class (susceptible or non-susceptible) and the majority-to-minority ratio did not exceed 20:1. Species were selected if they had at least 10 qualifying antibiotics, sufficient isolates (>50), and clinical relevance. Three species with sufficient isolates were excluded: Campylobacter jejuni (only three antibiotics qualified), Mycobacterium tuberculosis (only one antibiotic passed filtering), and Neisseria gonorrhoeae (incomplete AMRFinderPlus organism database coverage). In total, 107 species–antibiotic prediction tasks were defined across five species. Seven tasks have majority-to-minority ratios exceeding 10:1 (e.g. S. aureus linezolid 16.1:1, A. baumannii ceftriaxone 15.2:1), and their performance estimates should be interpreted with caution due to class imbalance. These thresholds were chosen to ensure sufficient minority-class representation for meaningful CV while avoiding extreme imbalance that would render F1 scores uninformative.

### Genomic annotation pipeline

Each assembled genome was processed through a 16-step annotation pipeline (Fig. 1, Supplementary Table S2) producing ~111 features per isolate-antibiotic pair. Detailed descriptions of each annotation step, including tool versions and parameters, are provided in Supplementary Methods S1 [17–25]. Point mutation features include locus-specific binary indicators (pf_has_gyrA, pf_has_gyrB, pf_has_parC, and pf_has_parE); per-locus mutation frequencies and resistance rates are in Supplementary Table S6.

Genotype–phenotype discordance was analyzed using variant-specific substrate matching (Supplementary Table S3) rather than broad drug-class matching. Expression-level features were compared between concordant and discordant cases. Full methodological details are provided in Supplementary Methods S1.

### Feature matrix construction and machine learning

Full hyperparameters for all classifiers are provided in Supplementary Table S7. No hyperparameter tuning was performed; default or literature-recommended values were used throughout. For each isolate-antibiotic pair, per-ARG annotation features were aggregated into a fixed-width numeric vector. Each antibiotic was mapped to its corresponding AMRFinderPlus drug class (e.g. ciprofloxacin to QUINOLONE), and “target” features were computed only from ARGs matching that drug class, while “genome-wide” features (total gene copies, total neighboring transposase count) were computed across all ARGs in the isolate. For numeric features, max and mean aggregations were used. When no target ARG was detected, target-level features were set to NaN and imputed with per-fold median during training.

MLST sequence types were encoded as 31 binary columns: the 30 most frequent STs per species plus an “st_other” column for all remaining STs. The final feature matrix contains one row per isolate-antibiotic pair with ~111 features.

Four classification algorithms were evaluated: LR (L2 penalty, balanced class weights) as a linear baseline, RF (300 trees, balanced class weights) as a non-linear ensemble robust to feature noise, XGB (100 trees, max_depth = 6, scale_pos_weight for class imbalance) as a gradient-boosted approach, and MLP (two hidden layers of 64 and 32 neurons, early stopping) to test non-linear feature interactions. All algorithms used a fixed random state (seed = 42) for reproducibility. Feature scaling (zero mean, unit variance) was applied within each CV fold to prevent data leakage.

All models were evaluated using five-fold stratified CV, with a separate model trained for each species–antibiotic combination. F1 score was used as the primary metric, with the default classification threshold of 0.5. Balanced accuracy, MCC, ROC–AUC, PR–AUC, and Brier score were also computed. To assess the impact of population structure on performance estimates, phylogeny-blocked CV was performed using pairwise ANI distances with average-linkage hierarchical clustering at 99.0%–99.5% ANI thresholds per species, followed by GroupKFold where all isolates in the same ANI cluster were assigned to the same fold.

A cumulative feature ablation study was performed using RF. Five configurations were evaluated, each adding feature groups incrementally: (a) ARG presence only (3 features), (b) plus expression features including promoter, RBS, and codon usage (26 features), (c) plus mobility features (38 features), (d) plus regulation features (56 features), and (e) the full model with all features (~111 features). For each configuration, mean F1 was computed as the macro-average over all species–antibiotic prediction tasks in the dataset, so that each task contributed equally regardless of isolate count. Learning curves were generated by training on stratified subsamples (n = 50 to 10 000). Feature importance was assessed using SHAP [26] TreeExplainer on RF. SHAP analysis used a stratified 80/20 train/test split held out separately from the CV folds used for performance estimation.

An RF model was trained on the 80% training fold (features standardized with StandardScaler) and SHAP values were computed on the 20% test fold, subsampled to 1000 isolates when the fold was larger, using the resistant class (Class 1). For each species–antibiotic task, feature importance was summarized as the mean absolute SHAP value across test samples. For each species, the five features with the largest mean absolute SHAP value averaged across all antibiotics tested for that species. No cross-feature or cross-antibiotic normalization was applied; reported SHAP magnitudes are therefore raw and should be compared only within, not across, species–antibiotic tasks.

## Results

### Dataset composition and prediction tasks

A total of 20 296 bacterial genomes with associated antimicrobial susceptibility phenotypes were analyzed across five species: *Salmonella enterica* (*n* = 10 070), *Escherichia coli* (*n* = 6668), *Klebsiella pneumoniae* (*n* = 1824), *Staphylococcus aureus* (*n* = 977), and *A. baumannii* (*n* = 757). *S. enterica* and *E. coli* originated predominantly from food-animal surveillance programs, national antimicrobial resistance monitoring system (NARMS), with poultry (34% and 33%), turkey (18% each), and swine contributing the majority of isolates. In contrast, *K. pneumoniae*, *S. aureus*, and *A. baumannii* were overwhelmingly from human clinical specimens (79%–89%). Geographically, 68.2% of isolates were from the USA, with Canada (9.2%) and Australia (2.4%) as the next most common origins.

**Figure 2:**
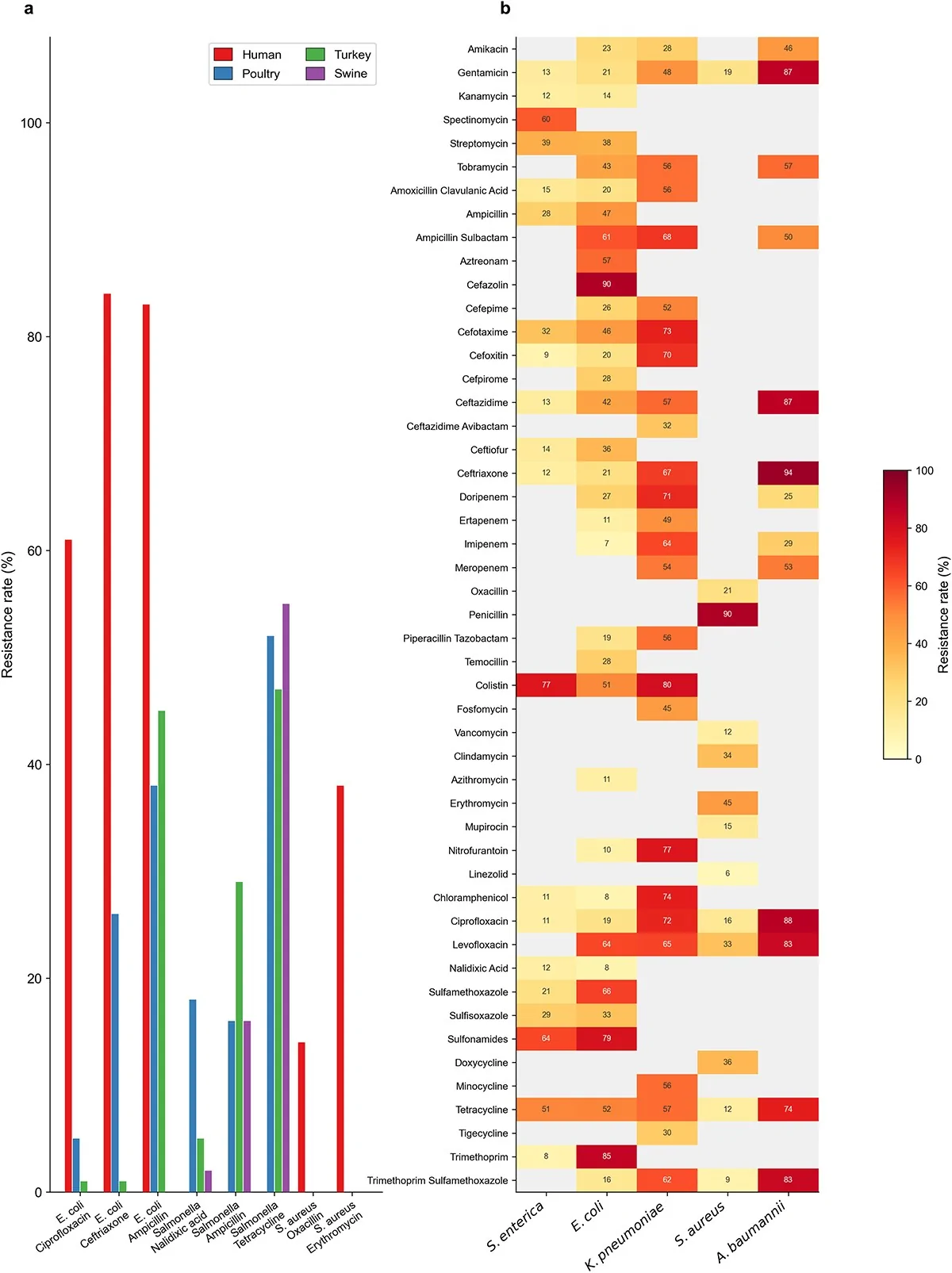
Dataset overview; (a) resistance prevalence by host source for selected antibiotics; (b) heatmap of resistance rates across species and antibiotics.

Human clinical *E. coli* had dramatically higher fluoroquinolone (61% versus 1%–5%) and cephalosporin (84% versus 1%–26%) resistance than food-animal isolates. Multi-locus sequence typing (MLST) analysis revealed near-complete phylogenetic separation by source: ST131, the pandemic extended-spectrum beta-lactamase (ESBL)-producing clone, comprised 34% of human clinical *E. coli* but was absent from poultry and turkey. In total, 107 species–antibiotic prediction tasks were defined, with beta-lactams constituting the largest group for gram-negative species, and *S. aureus* having a distinct gram-positive panel including oxacillin, vancomycin, clindamycin, linezolid, and erythromycin (Fig. 2).

### Gene-variant-specific discordance analysis

To avoid inflating discordance with gene-variant mismatches, each detected gene was evaluated against a curated mapping of substrate spectra (e.g. TEM-1 confers ampicillin but not cefepime resistance; aac(3)-IId confers gentamicin but not amikacin resistance; chromosomal blaEC was excluded as it does not confer resistance at basal expression [27]). With this matching strategy, isolate-antibiotic observations were classified into four categories: (i) ARG-explained resistance (variant-matched ARG present in resistant isolate), (ii) mutation-explained resistance (no variant-matched ARG but chromosomal point mutation detected), (iii) silent genes (variant-matched ARG present but isolate susceptible), and (iv) truly unexplained resistance (no ARG, no detected mutation, but resistant).

Variant-specific matching dramatically reduced apparent discordance: *E. coli* dropped from 33.3% (drug-class matching) to 9.3% because narrow-spectrum penicillinases (TEM-1) and intrinsic chromosomal AmpC (blaEC) were no longer counted as resistance determinants for cephalosporins and carbapenems. Point mutation accounting further reduced discordance by reclassifying quinolone-resistant isolates lacking ARGs. In *Salmonella*, 1820 quinolone-resistant isolates were explained by *gyrA*/*parC* mutations, reducing discordance from 4.8% to 3.3%, whereas in *E. coli*, 1483 were mutation-explained, reducing from 9.3% to 7.5%.

**Figure 3:**
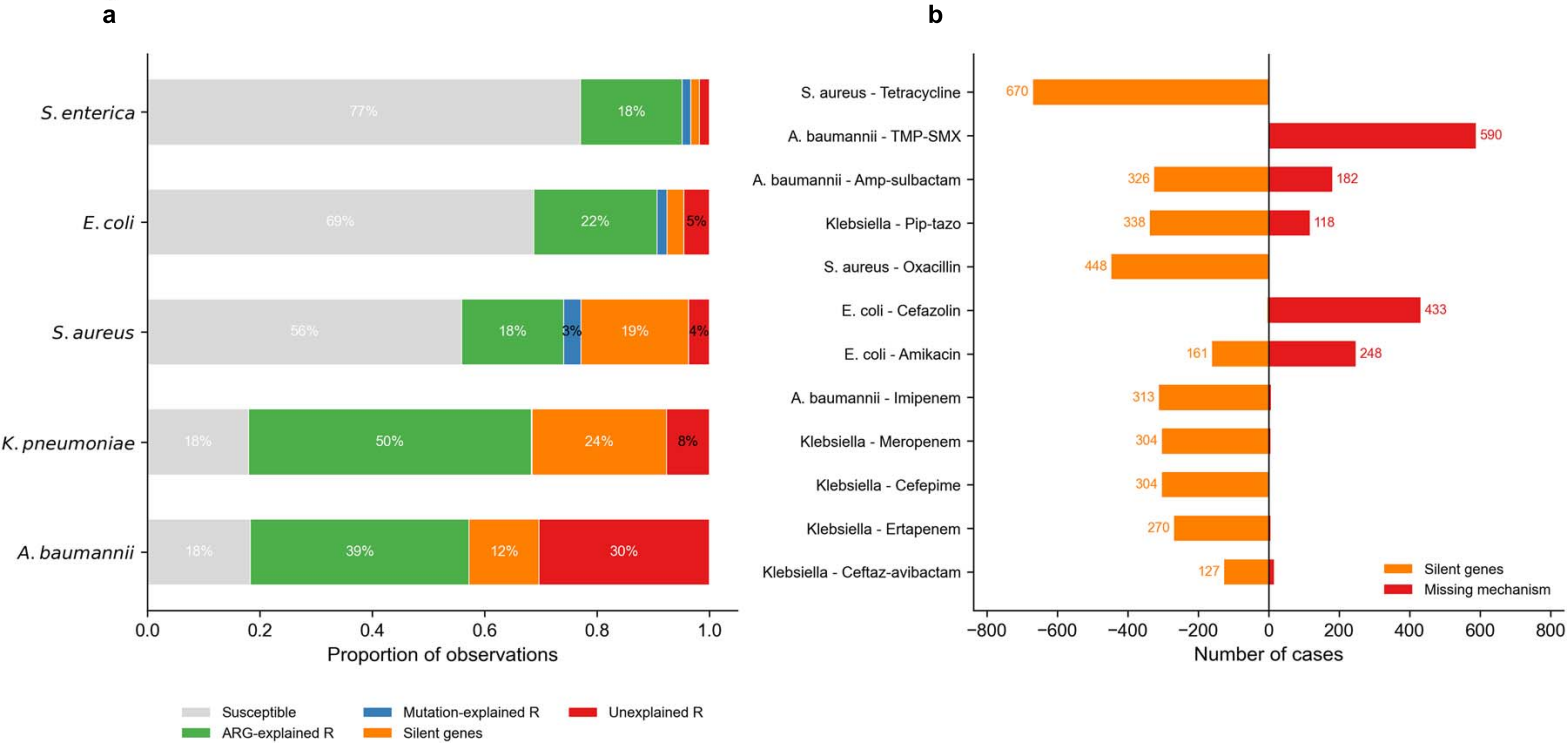
Genotype–phenotype discordance analysis; (a) breakdown of isolate-antibiotic observations into four categories (ARG-explained resistance, mutation-explained resistance, silent genes, and unexplained resistance) by species after variant-specific matching; (b) butterfly plot showing silent gene rates versus unexplained resistance rates per antibiotic.

After both corrections, true discordance ranged from 3.3% (*Salmonella*) to 42.7% (*A. baumannii*), comprising two mechanistically distinct types: silent genes (variant-matched ARG present but isolate susceptible) and unexplained resistance (no genetic determinant detected). Silent genes dominated in *K. pneumoniae* (23.9% of observations) and *S. aureus* (19.2%), while unexplained resistance was highest in *A. baumannii* (30.3%), partly reflecting limited mutation database coverage for this species—AMRFinderPlus identified only two point mutations (*gyrA_S81L* and *parC_E88K*) across our *A. baumannii* cohort—and the prominence of efflux- and porin-mediated mechanisms not captured by current annotation tools. Three distinct discordance patterns emerged across drug classes: missing-mechanism discordance dominated for quinolones and colistin, where resistance is chromosomal; true silent gene discordance dominated for *S. aureus* oxacillin (*mecA* present but SCCmec type affects expression), carbapenems *in K. pneumoniae* (carbapenemase present but expression varies), and *S. aureus* tetracycline (*tet(K)*/*tet(M)* variably expressed); and mixed discordance occurred for *E. coli* amikacin (161 silent +248 missing) and *A. baumannii* ampicillin-sulbactam (326 silent +182 missing) (Fig. 3).

### Expression features resolve silent gene discordance

For antibiotics with true silent genes, we compared expression-level features between concordant (ARG present, resistant) and discordant (ARG present, susceptible) isolates using only substrate-matched ARGs. For example, for gentamicin, only isolates carrying *aac(3)/ant(2″)* family genes were compared, excluding isolates carrying *aph(3′)* genes, which confer kanamycin but not gentamicin resistance. Silent ARGs consistently showed lower predicted translation initiation rates (30%–85% reduction, reflecting poor ribosome binding), 4%–14% points lower gene coverage (consistent with partial truncation, contig-edge effects, or insertion-sequence disruption of the gene call), and weaker promoter activity (8%–17% lower promoter linear discriminant function scores) than functional copies. The most dramatic difference was for chloramphenicol in *S. enterica*, where silent copies had 85% lower predicted translation initiation, indicating near-complete translational silencing of the ARG.

**Figure 4:**
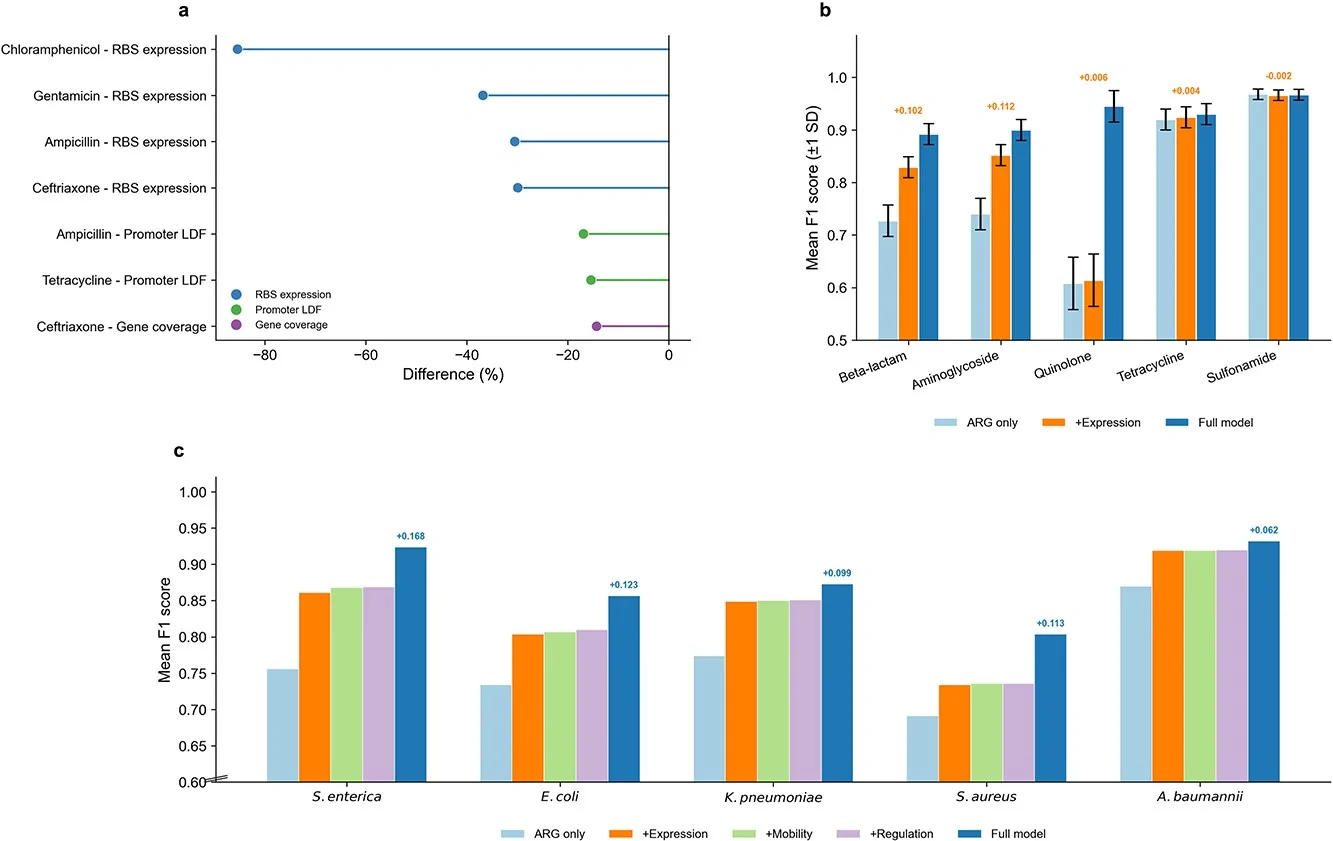
Feature ablation analysis; (a) expression-level differences between concordant and discordant cases (RBS expression, gene coverage, and promoter strength); (b) cumulative ablation F1 gains by drug class (bars represent mean F1 across five-fold stratified CV), (c) mean F1 across five feature configurations by species (note: *y*-axis is truncated at 0.7 to magnify differences).

Cumulative feature ablation confirmed these differences: expression features improved mean F1 by 0.07–0.11 over the ARG-only baseline for expression-dependent drug classes, while adding negligible gain for gene-sufficient antibiotics (Fig. 4).

### Cumulative feature ablation

The ablation study revealed that four feature groups contributed measurable predictive gain over the ARG-only baseline (Table 1): expression features (+0.07–0.11 F1 for beta-lactams and aminoglycosides), point mutations (+0.05–0.34 F1 for quinolones), gene identity/genomic context features (+0.02 F1), and population/MLST features (+0.012 F1). The modest but consistent contribution of population features reflects the fact that phylogenetic background captures latent carriage of co-selected resistance traits. In contrast, mobility features (IS elements, plasmid/prophage, and integrons) and regulation features (small RNA (sRNA), operon, and synteny) were dispensable (no measurable change in mean F1 when removed). The dispensability of mobility features is notable given the established role of IS elements in ARG expression [13, 28, 29] and suggests that expression features already capture the phenotypic consequence of IS-mediated activation.

**Table 1:** Feature group contributions to prediction performance.

Category	Feature groups	*N* features	Mean F1 gain	Role
Essential	ARG presence	3	Baseline^**a**^	Core signal
Essential	Expression (promoter, RBS, codon)	23	0.07	Resolves silent genes
Essential	Point mutations	8	0.05	Resolves missing mechanisms
Moderate	Gene identity (coverage, GC deviation)	9	0.02	Horizontal transfer signal
Dispensable	Mobility (IS, plasmid, integron)	15	−0.001	Redundant with expression
Dispensable	Regulation (sRNA, operon, synteny)	18	−0.001	No predictive value
Dispensable	Population (MLST)	35^**b**^	0.012	Lineage proxy, not generalizable

^a^ARG presence features define the starting model (mean F1 = 0.76 across species).

^b^Each species has exactly 31 ST columns (top 30 STs + st_other) plus four identity/coverage features.

Leave-one-out ablation (removing each feature group individually) confirmed that point mutation detail features had the largest marginal impact (mean F1 drop 0.016), followed by genomic context (0.005). Plasmid-context features showed measurable drops for cephalosporins, reconciling their high SHAP rankings with near-zero cumulative ablation gain (Supplementary Tables S4 and S5).

### Point mutations are essential for quinolone prediction

Across all five species, 69% of quinolone-resistant isolates had no acquired ARG; resistance was mediated by chromosomal point mutations. Adding point mutation features increased F1 from 0.41–0.90 to 0.92–0.98 for fluoroquinolones. Double mutants (*gyrA* + *parC*) showed 96.9% resistance rates versus 33.0% for gyrA-only in *E. coli* ciprofloxacin (Supplementary Table S6). The gyrA sequence alignment for *A. baumannii* is shown in Supplementary Fig. S1.

### Full model performance

With all features integrated, Random Forest (RF) achieved mean F1 of 0.804–0.932 across species (Table 2). RF significantly outperformed Logistic Regression (LR) (*P* = 4.3 × 10^−9^) and Multi-Layer Perceptron (MLP) (*P* = 5.3 × 10^−15^), and marginally outperformed Extreme Gradient Boosting (XGBoost) (*P* = 9.3 × 10^−3^). While XGBoost achieved marginally higher mean F1 for *E. coli* (0.861 versus 0.857) and *S. aureus* (0.818 versus 0.804), RF was selected for its consistency across all species and compatibility with SHAP TreeExplainer (Supplementary Fig. S2 and Supplementary Table S8). Species-level performance ranged from *A. baumannii* (mean F1 0.932) to *S. aureus* (0.804), correlating with dataset size and resistance mechanism diversity.

**Table 2:** Full model performance summary (Random Forest, five-fold stratified CV).

Species	N_antibiotics	Majority F1 ^a^	LR F1	RF F1	XGB F1	MLP F1	RF MCC	RF PR–AUC
*S. enterica* (*n* = 10 070)	20	0.154	0.908	0.924	0.920	0.905	0.903	0.935
*E. coli* (*n* = 6668)	35	0.205	0.836	0.857	0.861	0.792	0.804	0.902
*K. pneumoniae* (*n* = 1824)	26	0.601	0.846	0.873	0.865	0.753	0.702	0.928
*S. aureus* (*n* = 977)	13	0.073	0.770	0.804	0.818	0.605	0.753	0.873
*A. baumannii* (*n* = 757)	13	0.655	0.924	0.932	0.923	0.846	0.768	0.966

^a^F1 score of a naive classifier that predicts all isolates as the majority class (susceptible or resistant, whichever is more frequent) for each antibiotic, averaged across antibiotics per species. This represents the minimum performance achievable without any genomic information.

### Prediction failures

Nine species–antibiotic pairs achieved F1 <0.70 and represent the current limits of WGS-based prediction (Supplementary Table S1). These failures cluster into three mechanistically distinct causes: no database coverage for chromosomal or regulatory resistance (*E. coli* nitrofurantoin, F1 = 0.107; *K. pneumoniae* tigecycline, F1 = 0.513); complex mechanism requiring discrimination among multiple β-lactamase variants or combined enzyme–porin effects (four *E. coli* β-lactams, F1 = 0.429–0.669); and small sample size for rare resistant phenotypes (three *S. aureus* antibiotics, F1 = 0.462–0.632, *n* = 31–61). These causes are addressable by expanding mutation databases, encoding enzyme-variant substrate spectra and porin status, and conducting targeted surveillance of underrepresented phenotypes, respectively, supporting the broader argument that the residual genotype–phenotype gap is bounded and mechanistically traceable.

### SHAP feature importance

SHAP TreeExplainer analysis confirmed that the most important features per species directly reflect the dominant resistance mechanism (Table 3). For quinolones, the top SHAP feature was always a point mutation feature regardless of species (SHAP values 0.07–0.16), confirming that the same biological mechanism (target modification) is captured by the same computational feature across all organisms. For beta-lactams in Enterobacterales (*E. coli*, *S. enterica*, and *K. pneumoniae*), ARG presence features (has_target_arg, n_target_args) were the top global SHAP features, followed by guanine-cytosine (GC) deviation and codon adaptation features, reflecting the horizontally acquired nature of most beta-lactamases, while RBS thermodynamic features were more important in *S. aureus* (mecA expression) and *A. baumannii* (oxacillinase (OXA) expression regulation). For colistin, MLST and population features dominated in all three species with colistin data because resistance is predominantly chromosomal (mgrB inactivation, lipid A modification) without a well-characterized ARG, causing the model to rely on lineage-specific signatures rather than gene-level features. Gene count features (n_target_args, n_amr_genes) ranked in the top five for all species, confirming that gene dosage contributes to phenotype beyond simple presence/absence.

**Table 3:** Top five global SHAP features per species.

Rank	*S. enterica*	*E. coli*	*K. pneumoniae*	*S. aureus*	*A. baumannii*
1	has_target_arg	n_target_args	pf_n_quinolone_mut	pf_n_quinolone_mut	n_amr_genes
2	n_target_args	target_gc_dev	pf_has_quinolone_mut	pf_n_mutations	genome_gene_copies
3	genome_gc	has_target_arg	n_amr_genes	genome_gene_copies	genome_gc
4	target_n_plasmid	target_n_plasmid	genome_gene_copies	pf_has_*gyrA*	n_target_args
5	target_any_on_plasmid	arg_functional_score	pf_has_*parC*	n_amr_genes	target_gc_dev

Values are mean absolute SHAP from Random Forest TreeExplainer, computed on a held-out 20% test fold (Class 1, resistant) and averaged across all antibiotics tested for each species. Feature abbreviations: The pf_ prefix denotes point feature (chromosomal point mutation feature). has_target_arg = ARG presence for target drug class; n_target_args = number of target ARGs; n_amr_genes = total AMR genes; pf_n_quinolone_mut = number of quinolone point mutations; pf_has_quinolone_mut = quinolone mutation detected; pf_n_mutations = total point mutations; pf_has_*gyrA* = *gyrA* mutation detected; target_gc_dev = GC deviation from genome mean; genome_gene_copies = total gene copies across genome; genome_gc = genome GC content; target_n_plasmid = ARGs on plasmid; target_any_on_plasmid = any ARG plasmid-borne; arg_functional_score = composite ARG functionality score.

In Table 3, plasmid-context features (target_n_plasmid, target_any_on_plasmid) appear in the top-five SHAP rankings for *S. enterica* and *E. coli* even though Table 1 shows the near-zero ablation gain for mobility features. This reflects the different questions the two analyses answer: SHAP quantifies local per-prediction contribution, while ablation measures whether a feature group carries information that cannot be recovered from the remaining features. Plasmid context is largely redundant with ARG-presence features since ARGs on plasmids are already counted as such.

### Learning curve analysis

**Figure 5:**
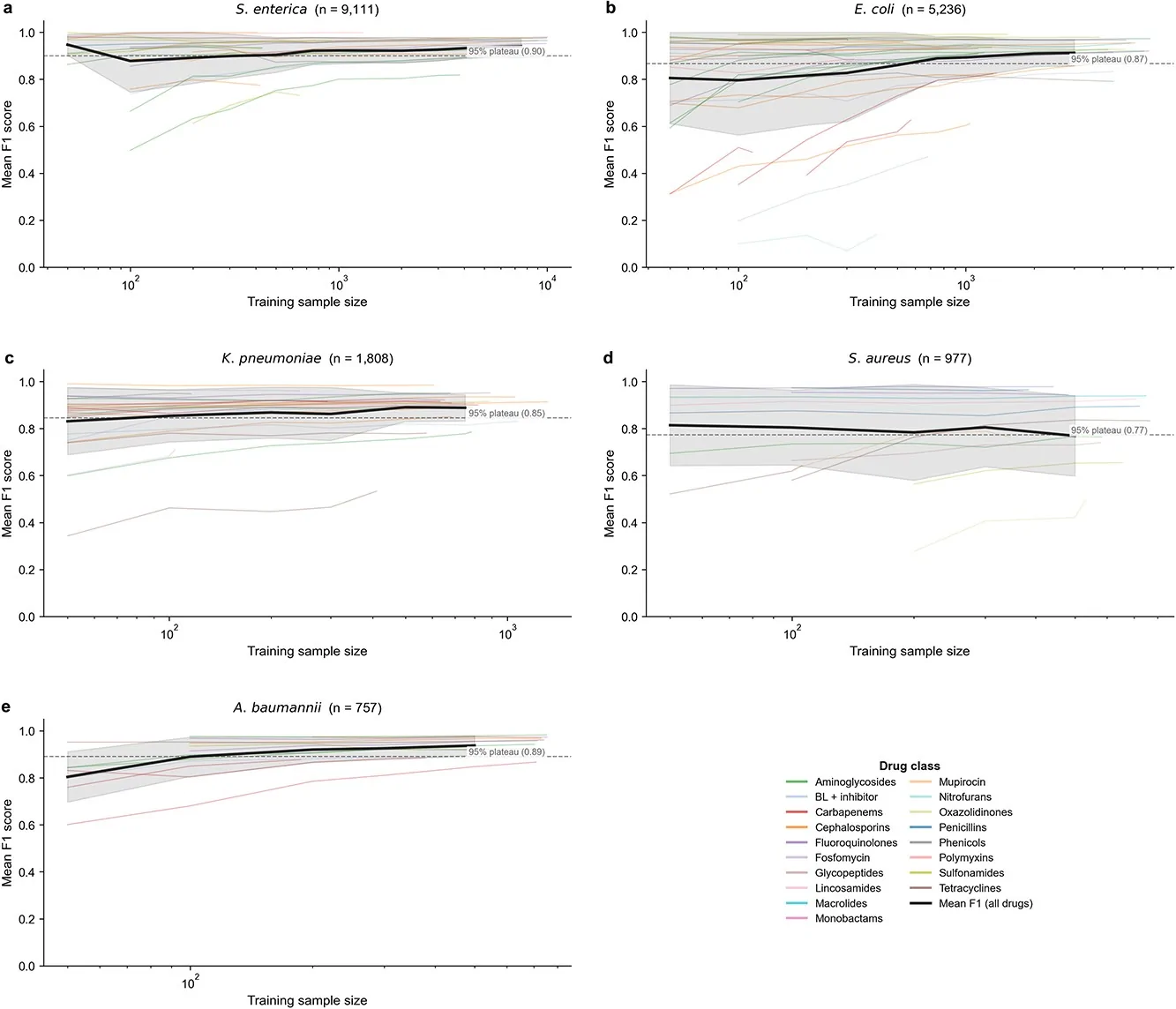
Learning curves; mean F1 scores (5× repeated five-fold stratified CV) at increasing training sample sizes (*n* = 50 to maximum), stratified by drug class and colored by species; single-gene resistance mechanisms (tetracyclines, sulfonamides) plateau by *n* = 200, while complex mechanisms (carbapenems, inhibitor combinations) require *n* > 1000.

Learning curve analysis using 5× repeated five-fold stratified cross-validation (CV) showed that 56% of prediction tasks reached a plateau, defined as 95% of the observed maximum F1 score, by *n* = 200 training samples. *S. enterica* showed the most rapid and stable convergence, whereas *E. coli* and *A. baumannii* improved more gradually as training size increased. *K. pneumoniae* showed relatively stable average performance, although some drug classes remained difficult to predict. *S. aureus* displayed greater variability and wider uncertainty within the available sample range (Fig. 5).

### Assembly fragmentation effects on expression features

Because expression features depend on the availability of intact upstream sequences, we assessed the impact of assembly fragmentation. In the training data (Illumina short-read assemblies), 17.3% of ARGs were located within 500 bp of a contig edge, and those within 100 bp had an 85.6% missing promoter rate compared to 41.2% for ARGs >1000 bp from edges. When promoters were detected, their strength (Linear Discriminant Function (LDF) scores) was similar regardless of contig position (4.05 versus 4.06), indicating that assembly fragmentation causes missing data rather than systematically biased values. The model handles missing expression features via median imputation. We note that median imputation was chosen for its simplicity and robustness; alternative strategies such as indicator variables for missingness were not explored but could potentially improve performance for edge-proximal ARGs. At a data level, long-read or hybrid assemblies (PacBio HiFi, Oxford Nanopore) would resolve the upstream-region truncation directly, although such platforms are not yet standard in routine AMR surveillance.

### Phylogeny-blocked cross-validation

To assess the impact of population structure on performance estimates [30], we performed phylogeny-blocked CV using average nucleotide identity (ANI)-based clustering (skani triangle, 99.0%–99.5% ANI threshold, and average-linkage hierarchical clustering). Cluster counts ranged from 20 (*S. aureus*) to 363 (*K. pneumoniae*) with singleton proportions from 2.5% (*S. aureus*) to 57% (*K. pneumoniae*). Overall, F1 dropped from 0.876 (five-fold stratified CV) to 0.753 (blocked CV, −0.123), confirming that stratified CV inflates performance when clonally related isolates appear in both training and test folds. The drop was drug-class-dependent: single-gene resistance generalized fully (ampicillin −0.003, tetracycline −0.002, and sulfisoxazole +0.002), while lineage-correlated predictions collapsed (*S. aureus* vancomycin 0.779 → 0.130, *S. enterica* colistin 0.994 → 0.504). Expression-dependent antibiotics showed intermediate drops. The blocked F1 of 0.753 still exceeds the ARG-only baseline (~0.60), confirming that expression features add value even across unseen lineages.

### External validation on independent Korean cohort

Pretrained RF models were applied to 141 independent ESBL *E. coli* isolates from South Korea [31]. Nalidixic acid achieved F1 = 0.931 (Matthews Correlation Coefficient (MCC) = 0.805), demonstrating strong generalization for point-mutation-based prediction. However, the model over-predicted resistance for 59% of susceptible isolates, reflecting higher resistance prevalence in the US-dominated training data (Supplementary Table S9).

Geographic hold-out validation (train USA, test non-USA) showed *E. coli* generalized best (mean F1 = 0.807 across 17 antibiotics), with ampicillin (0.958) and tetracycline (0.945) near-equivalent to CV. Performance drops for other species reflected genuine geographic differences in resistance prevalence, confirmed by human-clinical-only analysis (Supplementary Tables S10 and S11).

## Discussion

### A three-category conceptual framework

Our analysis of 107 species–antibiotic tasks reveals that AMR prediction failures are mechanistically heterogeneous, falling into three computationally separable categories—gene-sufficient, expression-dependent, and mutation-dependent—each requiring a distinct feature set. For gene-sufficient antibiotics (tetracyclines, sulfonamides, and ampicillin), gene presence alone achieves F1 >0.95, and additional features provide marginal value. These drug classes are mediated by dominant single genes (*tet(B)*, *sul1/sul2*, and *blaTEM-1*) whose presence reliably confers resistance.

For expression-dependent antibiotics (cephalosporins, aminoglycosides, and carbapenems), gene presence is necessary but not sufficient—silent genes with poor ribosome binding, truncation, and weak promoters are the primary source of false-positive predictions, and expression features improve F1 by 0.07–0.11. Importantly, expression features can be computed from assembled genome sequences without transcriptomic data, using RBS thermodynamic modeling (OSTIR), promoter prediction (BPROM), and codon usage analysis, making them suitable for routine clinical application.

For mutation-dependent antibiotics (quinolones, colistin, and vancomycin), resistance is primarily chromosomal, making gene catalogs fundamentally blind to the dominant mechanism, and point mutation features are essential. This framework explains why a “one-size-fits-all” approach cannot optimally predict all antibiotics. Beyond the dominant mechanism, prediction performance is also modulated by resistance prevalence, population structure, and dataset composition. The observed differences in expression-level features between silent and functional ARG copies—including 30%–85% lower translation initiation rates and 8%–17% lower promoter activity—correspond to known molecular mechanisms of gene silencing, providing biological validation that expression features capture real functional variation rather than statistical noise.

### Comparison with existing approaches

To directly benchmark against an existing phenotype prediction tool, we ran ResFinder 4.0 (with PointFinder for species with available databases) on all 20 296 assemblies and compared its phenotype predictions against the same antimicrobial susceptibility testing (AST) labels used for our ML models. Across the 79 species–antibiotic tasks where ResFinder produced predictions, it achieved mean F1 of 0.560–0.877 per species, comparable to our ARG-only baseline (0.708–0.887). The full model outperformed ResFinder for all five species: *E. coli* 0.748 → 0.878 (+0.130), *S. enterica* 0.877 → 0.926 (+0.048), *K. pneumoniae* 0.684 → 0.865 (+0.181), *S. aureus* 0.696 → 0.797 (+0.101), and *A. baumannii* 0.560 → 0.939 (+0.379). The *A. baumannii* gain is particularly large because ResFinder lacks a PointFinder database for this species and therefore cannot detect chromosomal point mutation-based resistance (e.g. ciprofloxacin F1 = 0.003). CARD RGI was not separately benchmarked as it does not emit antibiotic-level phenotype calls comparable to ResFinder and draws on the same resistance determinant classes. Per-species summaries are in Supplementary Table S12; per-antibiotic results in Supplementary Table S12b.

Several studies have applied ML to WGS-based AMR prediction. Drouin *et al*. [8] used *k*-mer features with set-covering machines; Nguyen *et al*. [9] applied XGBoost to pangenome features; VanOeffelen *et al*. [11] systematically benchmarked ML methods across 67 species–drug datasets; and Hu *et al*. [12] evaluated state-of-the-art tools on 78 datasets. Direct comparison with *k*-mer-based methods was not feasible as they require raw reads rather than assemblies; however, Hu *et al*. reported comparable gene-detection-level performance.

Our approach differs from these prior studies in three key respects. First, we explicitly encode expression-level features that distinguish functional from silent genes, directly addressing the systematic false-positive problem in gene-catalog approaches. Second, we use variant-specific discordance analysis rather than drug-class-level matching, revealing that much apparent discordance is a methodological artifact (e.g. *E. coli* discordance dropping from 33% to 9.3%). Third, we provide a SHAP-based mechanistic interpretation that identifies which features drive predictions for each drug class, enabling biologically informed tool development.

F1 score was chosen as the primary evaluation metric because it penalizes both false positives (over-prediction of resistance leading to unnecessary broad-spectrum therapy) and false negatives (missed resistance leading to treatment failure), both of which are clinically consequential. We used predict_proba with a fixed 0.5 classification threshold for all models. To ensure robustness, we report threshold-independent metrics (Receiver Operating Characteristic Area Under the Curve (ROC–AUC), Precision-Recall Area Under the Curve (PR–AUC) and class-imbalance-robust metrics (MCC, balanced accuracy) alongside F1 for all analyses (Supplementary Tables S4, S5, and  S8). Our inclusion criteria (minority class ≥30, ratio ≤20:1) exclude extreme imbalance, and balanced class weights were used in all classifiers.

### Implications for deployment

The learning curve results have practical implications for implementing WGS-based AMR prediction in surveillance: for species and antibiotics with well-characterized single-gene resistance mechanisms, prediction systems can be deployed with relatively small training datasets. For mechanistically diverse species like *E. coli*, training data must be pooled across institutions and geographies to capture the full spectrum of resistance mechanisms. Rare phenotypes (linezolid, tigecycline) require deliberate enrichment of resistant isolates in training sets. Five-fold stratified CV reflects realistic performance within the training surveillance population; blocked CV defines the lower bound for novel lineages.

### Limitations and future directions

Our expression features (BPROM, OSTIR, and Codon Adaptation Index (CAI)) are computational predictions, not experimentally validated. BPROM was trained on *E. coli* sigma-70 promoters and may have reduced accuracy for other species. OSTIR predictions of translation initiation rates have been validated against reporter assays but not specifically for ARG contexts. Despite potential systematic biases, the consistent association between low predicted expression and phenotypic susceptibility across five species (Fig. 4a) supports biological relevance, though the potential impact on model generalizability warrants caution. RNA-seq or Ribo-seq validation is recommended in future work.

Several limitations should be noted. First, our approach is database-dependent: resistance mechanisms not in AMRFinderPlus (nitrofurantoin *nfsA/nfsB* mutations, tigecycline *ramA/ramR* regulatory mutations) are invisible to the pipeline, and the approach cannot discover novel resistance mechanisms.

Second, although we performed phylogeny-blocked CV, the choice of ANI threshold (99.0%–99.5%) and clustering method (average-linkage hierarchical) represents one of many possible approaches, and the optimal threshold may vary by species. The severe performance drops observed for some antibiotics (*S. aureus* vancomycin, *S. enterica* colistin) indicate that lineage confounding remains a concern for chromosomally-mediated resistance, and further work is needed to establish the most informative phylogenetic granularity for CV.

We acknowledge that the causal relationship between reduced gene coverage and gene silencing remains unclear—the observed lower coverage could reflect either true biological truncation or technical artifacts from incomplete assembly, annotation errors, or contig-edge effects. Short-read assemblies are particularly susceptible to fragmentation at repetitive IS elements flanking ARGs. Distinguishing biological from technical causes would require long-read sequencing data.

Third, external validation on the Korean ESBL cohort revealed systematic over-prediction, likely reflecting geographic differences in resistance prevalence. Calibration to local epidemiology may be necessary for clinical deployment.

Fourth, the feature matrix contains ~111 features per isolate-antibiotic pair, organized in 12 biologically motivated subgroups that Table 1 collapses to seven higher-level categories for the ablation analysis.

Finally, permeability-based resistance (porin loss, efflux overexpression) remains intrinsically difficult to predict from genome sequence alone. Despite these limitations, the framework provides a reproducible, mechanistically interpretable basis for evaluating WGS-based AMR prediction across diverse species and drug classes.

**Key Points**

- Variant-specific substrate mapping reduces apparent genotype–phenotype discordance from 33.3% to 9.3% in *Escherichia coli*, revealing that a substantial fraction of reported discordance reflects substrate misattribution rather than true gene silencing.
- Sequence-based expression potential features distinguish functional from silent resistance genes without transcriptomic data, improving F1 by 0.07–0.11 for beta-lactams and aminoglycosides where silent gene rates reach 27%–36%.
- Antimicrobial resistance mechanisms fall into three categories—gene-sufficient, expression-dependent, and mutation-dependent—each requiring distinct computational features, providing a mechanistic framework for when and why whole-genome sequencing-based prediction fails.
- Phylogeny-blocked cross-validation confirms that single-gene predictions generalize across unseen lineages (F1 drop <0.01), while the full model retains substantial predictive value even on novel lineages (blocked F1 = 0.753, exceeding gene-presence-only baseline of ~0.60).

## Supplementary Material

Supplementary_material_bbag451

## Data Availability

All genome assemblies are publicly available from the NCBI SRA and BV-BRC databases. Feature matrices, pretrained models, and per-ARG annotation summaries are deposited at https://github.com/tatsu1207/AMR.
